# Treatment with a corticotrophin releasing factor 2 receptor agonist modulates skeletal muscle mass and force production in aged and chronically ill animals

**DOI:** 10.1186/1471-2474-12-15

**Published:** 2011-01-14

**Authors:** Richard T Hinkle, Frank R Lefever, Elizabeth T Dolan, Deborah L Reichart, Janice M Zwolshen, Timothy P Oneill, Kris G Maloney, John P Mattson, Leonardo F Ferreira, Timothy I Musch, David C Poole, Robert J Isfort

**Affiliations:** 1 Research Division, Procter & Gamble Company, Mason, OH, USA; 2 Department of Health and Exercise Science, Gustavus Adolphus College, St. Peter, MN, USA; 3 Departments of Kinesiology and Anatomy & Physiology, Kansas State University, Manhattan, KS, USA

## Abstract

**Background:**

Muscle weakness is associated with a variety of chronic disorders such as emphysema (EMP) and congestive heart failure (CHF) as well as aging. Therapies to treat muscle weakness associated with chronic disease or aging are lacking. Corticotrophin releasing factor 2 receptor (CRF2R) agonists have been shown to maintain skeletal muscle mass and force production in a variety of acute conditions that lead to skeletal muscle wasting.

**Hypothesis:**

We hypothesize that treating animals with a CRF2R agonist will maintain skeletal muscle mass and force production in animals with chronic disease and in aged animals.

**Methods:**

We utilized animal models of aging, CHF and EMP to evaluate the potential of CRF2R agonist treatment to maintain skeletal muscle mass and force production in aged animals and animals with CHF and EMP.

**Results:**

In aged rats, we demonstrate that treatment with a CRF2R agonist for up to 3 months results in greater extensor digitorum longus (EDL) force production, EDL mass, soleus mass and soleus force production compared to age matched untreated animals. In the hamster EMP model, we demonstrate that treatment with a CRF2R agonist for up to 5 months results in greater EDL force production in EMP hamsters when compared to vehicle treated EMP hamsters and greater EDL mass and force in normal hamsters when compared to vehicle treated normal hamsters. In the rat CHF model, we demonstrate that treatment with a CRF2R agonist for up to 3 months results in greater EDL and soleus muscle mass and force production in CHF rats and normal rats when compared to the corresponding vehicle treated animals.

**Conclusions:**

These data demonstrate that the underlying physiological conditions associated with chronic diseases such as CHF and emphysema in addition to aging do not reduce the potential of CRF2R agonists to maintain skeletal muscle mass and force production.

## Background

### Aging and frailty

Skeletal muscle mass and function is reduced during aging resulting in frailty and weakness in elderly individuals, thereby markedly increasing the risk of disability and loss of functional capacity [1]. The loss of skeletal muscle mass and function with aging results in decreased reserves of skeletal muscle which, when combined with acute illness, often results in decreased mobility and quality of life [1]. Current concepts regarding the mechanisms that cause the loss of skeletal muscle mass and function during aging include some combination of inactivity, nutritional imbalance, cumulative damage, metabolic alterations resulting in increased catabolism and decreased anabolism, hormone loss (including growth hormone, IGF-1, androgens and estrogen), increased levels of cachectic cytokines and loss of muscle regeneration potential [2-8].

Animals as well as humans suffer from aging related loss of skeletal muscle function. Several animal models of aging related muscle loss exist, with one of the most studied models being the 24 month old aging rat model [9-15]. Aging F344 rats demonstrate many of the hallmarks of human aging related muscle loss and have been used to evaluate the potential of several compounds, including beta adrenergic agonists and ACE inhibitors, to prevent or reverse aging related muscle loss [10,12-14,16-19].

### Emphysema and Muscle Function

Chronic hypercapnia is associated with a poor prognosis in patients afflicted with chronic obstructive pulmonary disease (COPD) [20,21]. The mechanisms leading to chronic hypercapnia are not fully known although it is believed that inspiratory muscle fatigue and/or weakness leads to CO_2 _retention and ultimately respiratory failure. Indeed, Roussos demonstrated that hypercapnic COPD patients reach a critical zone of fatigue by requiring 2-3 times the transdiaphragmatic pressure that normocapnic patients produce during breathing at rest [22]. In COPD patients, respiratory muscle weakness and diaphragm fiber atrophy decreases respiratory muscle reserves increasing muscle fatigability/weakness thereby predisposing the patient to chronic hypercapnia [23]. The changes in diaphragm muscle that occur during EMP include muscle fiber shortening by loss of sarcomeres in series [24,25], increase in cross-sectional area of type I and II fibers [26,27], atrophy [28,29] and loss of oxidative enzyme capacity [30]. While the adaptive changes in diaphragm muscle are complex, ultimately EMP augments the energetic requirements of respiratory muscles which, concomitant with EMP-induced reductions in muscle mass, contributes to diaphragm weakness, increased fatigability and overall dysfunction.

In EMP, the diaphragm is not the only skeletal muscle to develop weakness. In humans and animals with EMP, changes in peripheral skeletal muscles have been described including atrophy [27,31], reduced myocyte cross-sectional area [27,31], loss of type IIB fibers [27], increased fatigability [32,33], lipofuscin inclusions [33] and increased antioxidant enzyme levels [33]. Thus in EMP, overall skeletal muscle function is altered and therapies with the potential to improve skeletal muscle function may have beneficial effects.

### CHF and muscle wasting

Skeletal muscle wasting associated with congestive heart failure is part of a general wasting syndrome associated with CHF known as cardiac cachexia [34]. Cardiac cachexia typically affects about 20% of CHF patients with the cachectic CHF patients showing reduced muscle mass and strength resulting in reduced exercise capacity and impaired activities of daily living when compared to noncachectic CHF patients [34]. The mechanism(s) responsible for CHF associated skeletal muscle wasting are at present unknown although it has been proposed that alterations in catabolic cytokines/hormones such as TNF-α, IL-6 and cortisol result in CHF mediate skeletal muscle wasting [34-36]. In order to study CHF associated skeletal muscle wasting, rodent CHF models have been extensively utilized with one of the most relevant model being the experimental infarct model [37-45]. Clinical and animal studies have demonstrated that various pharmacological agents such as human growth hormone, angiotensin II receptor antagonists and ghrelin can reduce the level of muscle wasting associated with CHF [37-39]. Thus, pharmacologically- induced increases in skeletal muscle mass and strength in CHF patients with skeletal muscle wasting may improve skeletal muscle strength and function.

### CRF2R activation and muscle function

Recently, we have demonstrated that activation of the CRF2R reduces acute skeletal muscle atrophy resulting from disuse, nerve damage, corticosteroid treatment and causes hypertrophy of non-atrophying skeletal muscle in healthy animals [46-48]. CRF2R activation also reduces skeletal muscle wasting associated with two chronic disease conditions, cancer and muscular dystrophy [49-51]. We have not yet investigated if activation of the CRF2R will maintain skeletal muscle mass and force production in animals that have an underlying chronic condition that results in muscle wasting. Therefore, we have utilized the aging rat model, the emphysematous hamster model and the rat CHF model to determine if activation of the CRF2R will maintain skeletal muscle mass and force production in chronically ill and aged animals. The results of these investigations are presented in this report.

## Methods

The CRF2R selective agonist PG873637 was synthesized at Procter & Gamble as described previously [52]. Twenty eight day rat osmotic minipumps were purchased from Alza Corporation (Palo Alto, CA). PG873637 was administered using twenty eight day osmotic minipumps at a dose of 100 ug/kg/d, which we have shown provides maximal CRF2R agonist activity over the 28 day dosing period (unpublished data). All animal studies described in this report were conducted in compliance with the US Animal Welfare Act, the rules and regulations of the State of Ohio Departments of Health, Procter & Gamble's policy on research involving animals with strict oversight for care and welfare and were approved by the Procter & Gamble Institutional Animal Care and Use Committee. In addition, the protocols used in the hamster emphysema studies were approved by the Gustavus Adolphus College and the Kansas State University Institutional Animal Care and Use Committees. In all respects, they conform to the Guide for the Care and Use of Laboratory Animals published by the US National Institutes of Health (NIH Publication No. 8523, revised 1985).

### Aging rat model

Between 8 (time 0 and 1 month treatment) and 16 (3 months treatment) 24 month old F344 female rats per treatment group, purchased from the Harlan (Indianapolis, IN), were double-housed and acclimatized to the conditions of the facility for approximately 1 week before use. Rats had access to lab chow and water ad libitum and were subjected to standard conditions of humidity, temperature and a 12 hour light cycle. Rats were divided into five treatment groups: Time 0 - 24 months old rats prior to treatment; 1 month vehicle - 24 month old rats at initiation of treatment and treated with vehicle for 1 month (25 months old at time of harvest); 1 month PG873637 - 24 month old rats at initiation of treatment and treated with 100 ug/kg/day PG873637 for 1 month (25 months old at time of harvest); 3 month vehicle - 24 month old rats at initiation of treatment and treated with vehicle for 3 months (27 months old at time of harvest); 3 month PG873637 - 24 month old rats at initiation of treatment and treated with 100 ug/kg/day PG873637 for 3 months (27 months old at time of harvest). A larger number of rats were included in the 3 month treatment group (compared to time 0 and 1 month) since loss of rats due to their extreme age was anticipated. Rats were anesthetized with isoflurane and Alza 28-day rat minipumps containing either vehicle or PG873637 were implanted subcutaneously in the midscapular region of the back. Pumps were replaced every 28 days until the end of the study. At the specified times, rats were sacrificed and leg muscles were removed and evaluated.

### Hamster emphysema model

Male 3 month old Syrian Golden hamsters (120-130 g) were purchased from Harlan (Indianapolis, IN) and were housed using standard conditions including ad libitum access to lab chow/water, standard conditions of humidity/temperature and a 12 hour light cycle. Hamsters were divided randomly into sham (*n *= 14) and emphysema (EMP) conditions (*n *= 24). Under deep ketamine/xylazine anesthesia (150/7.5 mg/kg im), either saline (0.3 ml/100 g body wt) (Sham) or porcine elastase (25 IU/100 g body wt in 0.3 ml of normal saline, Sigma Chemical, St. Louis, MO) (EMP) was instilled intratracheally as described previously (Mattson and Poole, 1998). Four months following instillation, hamsters were assigned randomly to the following experimental groups: (6 Sham-Vehicle + 13 EMP-Vehicle) + (6 Sham-Treatment + 12 EMP-Treatment). The CRF2R selective agonist (PG873637) (Treatment) or physiological saline (Vehicle) was delivered (100 μg/kg/d) via 28-day osmotic mini-pumps implanted in the midscapular region starting at month 4 and changed monthly for 5 months. Following 5 months of treatment, the lungs, heart, costal diaphragm and EDL muscle were removed and evaluated. To determine disease severity, a saline displacement technique was used to measure excised lung volume and the right ventricle (RV) was dissected and weighed as described previously [53].

### Rat CHF model

#### Myocardial infarction (MI) surgery

Surgically-induced myocardial infarctions were performed as described previously [54]. Male 3 month old Lewis rats were purchased from the Charles River Laboratories and double-housed and acclimatized to the conditions of the facility for approximately 1 week before use. Rats had access to lab chow and water ad libitum and were subjected to standard conditions of humidity, temperature and a 12 hour light cycle. Rats were administered an injection of an analgesic [buprenorphine, 0.05 mg/kg, intramuscular (im)] and an antibiotic (amoxicillin, 125 mg/kg, sc) prior to surgery. Rats were then anesthetized with 2-5% isoflurane. Once anesthetized, an endotracheal tube was placed in the trachea of each rat and a respirator used to mechanically ventilate the rat and control the flow of isoflurane. Next, electrocardiogram (ECG) electrodes were attached and the ECG monitored. Animals were then injected with lidocaine 2% (0.1 ml/rat, im) to stabilize heart rhythm. The surgical site was prepared by shaving the left thoracic area, disinfecting the skin with surgical scrub, and wiping with 70% isopropanol. A left thoracotomy was performed at the fifth intercostal space to the left of the sternum, the heart carefully exposed, the pericardium opened, and the left anterior descending coronary artery (LAD) located. One or more 5-0 ligatures were permanently tied around the LAD, and the heart replaced in the chest. The heart rate and rhythm was monitored for approximately 10 minutes, and supplemental lidocaine administered as needed. Once cardiac rhythm stabilized, the lungs were maximally inflated and the incision closed with suture. Mechanical ventilation was discontinued when the animal was able to breathe unassisted. The endotracheal tube was removed when the animal exhibited a swallow reflex. Animals were then allowed to recover from anesthesia in heated cages and visually monitored for signs of distress for approximately 1 hour. Rats received additional doses of buprenorphine (0.05 mg/kg, im) daily for 2 days following surgery.

#### Echocardiography evaluation

Previously infarcted male Lewis rats were anesthetized with isoflurane. The chest was shaved, and short-axis and long-axis views of the left ventricle were captured. Mitral and aortic Doppler flows were also taken to estimate cardiac output and LV compliance. After the examination, the animals were returned to their cages to recover from anesthesia.

#### Infarct screening by echocardiography

Myocardial infarct size was determined by echocardiography. Approximately 1-2 weeks post-ligation, rats were screened for infarct size. Rats were grouped based on clinical presentation as follows: group 1 - small infarct, scar area circumference of 25% or less and a FAC < 30%; group 2 - medium to large infarct, scar area circumference of >33%, FAC < 30%, mitral regurgitation and LV dilation; group 3 - very large infarct, scar area circumference of >40%, FAC < 30%. Rats were randomized into treatment groups based on infarct size; only rats having a large myocardial infarct (40% or greater) were used that met the following inclusion criteria - large scar, diastolic dilation and decreased fractional area contraction (FAC) as follows. Sham surgery consisted of the same surgery without ligation of the coronary artery. Drug treatment (vehicle or active) was started 6 weeks post surgery for both myocardial infarcted and sham surgery rats.

#### Post-mortem infarct analysis

The intact LV myocardium and scar were splayed, pressed between 2 pieces of plastic and traced on clear vinyl. The tracings were scanned into image files, and analyzed for infarct size as a percent of the intact LV using a custom Alpheion macro by T. DuFresne, Procter & Gamble.

#### Osmotic minipump implantation

Between 7 (sham) and 10 (myocardial infarcted) rats per treatment group were anesthetized with isoflurane and Alza 28 day rat minipumps were implanted in the midscapular region. A larger number of rats were included in the myocardial infracted group since loss of rats due to their congestive heart failure was anticipated. Pumps were replaced every 28 days until the end of the study. PG873637 was administered at a dose of 100 ug/kg/d.

### Leg muscle function analysis

At the end of each of the studies, animals were anesthetized using isoflurane, the right leg was shaved, and the right extensor digitorum longus (EDL) and/or soleus muscles exposed. Silk sutures were tied to the proximal and distal tendons of the EDL and/or soleus muscles and the EDL and/or soleus muscles were removed, tendon-to-tendon. The muscles were placed into a plexiglass chamber filled with Ringer solution (137 mM sodium chloride, 24 mM sodium bicarbonate, 11 mM glucose, 5 mM potassium chloride, 1 mM magnesium sulfate, 1 mM sodium phosphate, 0.025 mM tubocurarine - all at pH 7.4, constantly oxygenated with 95% oxygen/5% carbon dioxide) and maintained at 25°C. Muscle were aligned horizontally between a servomotor lever arm and a stainless steel fixed-post (Aurora Scientific Inc., model 6650LR) and field stimulated by pulses transmitted between two platinum electrodes placed longitudinally on either side of the muscle. Square wave pulses (0.2 ms duration) generated by a personal computer with a Labview board (Model PCI-MIO-16E-4, Labview Inc., Austin, TX, USA) were amplified (Acurus Power Amplifier Model A250, Dobbs Ferry, NY, USA) to increase and sustain current intensity to a sufficient level to produce a maximum isometric tetanic contraction. Stimulation voltage and muscle length (Lo) were adjusted to obtain maximum isometric twitch force. Maximum tetanic force production (Po) was determined from the plateau of a frequency-force relationship

### Statistical analysis

Statistical analysis was performed using an ANCOVA model with treatment effect and starting weight as the covariates. Pair wise comparisons for all end-points were generated using least-square means (SAS, Cary, North Carolina), adjusted for unequal sample sizes and starting weight.

## Results

### Aging rat model

#### Evaluation of muscle mass and Po following CRF2R agonist treatment

Evaluation of absolute EDL muscle mass and force in 24, 25 and 27 month old female F344 rats did not show a significant difference in absolute EDL mass with age but did demonstrate a significant decrease in absolute force (Po) at 27 months of age (13% decrease compared to 24 months of age) (Table 1). There were no statistically significant aging-related differences in absolute soleus muscle mass or Po (Table 1). An evaluation of aging related differences in the relative EDL and soleus muscle mass and Po demonstrated greater relative EDL and soleus muscle mass with age; there were no differences in relative EDL Po with age while relative soleus Po was greater at 25 months of age compared to 24 months of age but this difference was not observed at 27 months of age. The difference in relative muscle mass/Po compared to absolute muscle mass/Po is mainly driven by the decrease in body mass observed with aging.

**Table 1:** Effects of 1 month or 3 months of treatment with either vehicle or PG873637 on female 24 month old F344 rat body mass, muscle mass, muscle absolute force (Po).

	Time 0	1 month vehicle	1 month PG873637	3 month vehicle	3 month PG873637
Number of animals	8	8	7	14	16

Final body mass (grams)	281.6 (8.1)	269.4 (5.0)	252.0# (4.2)	256.9# (6.7)	252.0# (5.1)

EDL mass (milligrams)	91.9 (1.0)	89.0 (1.9)	101.2*# (2.1)	89.4 (1.5)	101.3*# (4.2)

EDL mass/body mass (milligram/gram)	0.33 (0.00)	0.34 (0.01)	0.40*# (0.01)	0.35# (0.01)	0.40*# (0.01)

EDL Po (milliNewtons)	2025.2 (18.7)	1965.6 (36.6)	2199.3*# (59.3)	1761.4# (56.3)	2004.6^(*p = 0.06)^ (114.2)

EDL Po/body mass (milliNewton/gram)	7.23 (0.09)	7.42 (0.11)	8.68*# (0.30)	6.86 (0.19)	7.86* (0.40)

Soleus mass (milligrams)	79.8 (2.2)	82.6 (2.1)	89.0# (3.2)	81.3 (2.0)	88.3 (4.0)

Soleus mass/body mass (milligram/gram)	0.28 (0.01)	0.31# (0.01)	0.35*# (0.01)	0.32# (0.01)	0.35^(*p = 0.06)^# (0.01)

Soleus Po (milliNewtons)	1188.3 (24.6)	1244.5 (28.6)	1362.5*# (45.3)	1128.1 (45.2)	1278.2 (81.3)

Soleus Po/body mass (milliNewton/gram)	4.25 (0.08)	4.71# (0.10)	5.37*# (0.20)	4.39 (0.16)	5.03^(*p = 0.06)^# (0.29)

All data are given as the mean with the standard error of the mean in parenthesis. * - statistically significant difference (p < 0.05) versus the appropriate vehicle control; # - statistically significant difference (p < 0.05) versus Time 0.

Evaluation of age-matched CRF2R agonist (PG873637) treated versus vehicle treated rats resulted in greater absolute EDL mass (+14%/+13%) and Po (+12%/+14%^p = 0.06^) and relative EDL mass (+18%/+14%) and Po (+17%/+15%) with 1 and 3 months of PG873637 treatment when compared to the appropriate vehicle control (data given as 1 month PG873637 over vehicle % increase/3 months PG873637 over vehicle % increase). Absolute soleus mass was greater (+8%/+9%) although this difference was not statistically significant; absolute soleus Po was greater (+9%/+13%) although only the 1 month difference was statistically significant. Relative soleus mass (+12%/+9%^p = 0.06^) and Po (+14%/+15% ^p = 0.06^) was greater with 1 and 3 months (respectively) of PG873637 treatment when compared to the appropriate vehicle control (Table 1).

### Hamster emphysema model

#### EMP induction

As can be seen in Table 2, a large increase in lung volume for both vehicle and PG873637 treated EMP hamsters [EMP-vehicle (+141%) and EMP-PG873637 (+113%)] was observed relative to the vehicle and PG873637 treated non-emphysemic control (Sham) hamsters; lung volume-to-body weight ratios demonstrated a similar change [EMP-vehicle (+136%) and EMP-PG873637 (+154%)]. Lung volume was similar within the EMP and Sham groups with and without PG873637 treatment. Analysis of the heart indicated that right ventricle weight was similar in both vehicle and PG873637 treated EMP hamsters [EMP-vehicle (+26%) and EMP-PG873637 (+46%)] when compared to the vehicle and PG873637 treated Sham hamsters; RV mass-to-body weight ratios demonstrated a similar difference [EMP-vehicle (+28%) and EMP-PG873637 (+67%)]. RV mass was similar within the EMP and Sham groups with and without PG873637 treatment.

**Table 2:** Effect of 5 months of treatment with either vehicle or PG873637 on female sham or emphysemic (EMP) hamster final body mass, lung volume, right ventricular (RV) mass, muscle mass and muscle absolute force (Po).

	Sham vehicle	Sham PG873637	EMP vehicle	EMP PG873637
Number of animals	6	6	9	11

Final body mass (grams)	148.7 (7.6)	162.3 (9.0)	149.8 (4.1)	142.2 (4.4)

Lung volume (milliliter)	2.3 (0.2)	2.4 (0.2)	5.4# (0.4)	5.2# (0.6)

Lung volume/body mass (milliliter/gram)	0.02 (0.00)	0.02 (0.00)	0.04# (0.00)	0.04# (0.01)

RV mass (milligrams)	115.7 (4.1)	109.6 (4.0)	145.5# (6.3)	159.7# (9.7)

RV mass/body mass (milligram/gram)	0.78 (0.06)	0.69 (0.02)	0.99# (0.06)	1.15# (0.08)

EDL mass (milligrams)	32.6 (2.2)	46.0* (3.8)	33.0 (1.5)	35.7 (1.4)

EDL mass/body mass (milligram/gram)	0.22 (0.01)	0.28* (0.02)	0.22 (0.01)	0.25* (0.01)

EDL Po (milliNewtons)	832.5 (45.1)	1229.2* (36.1)	837.6 (29.0)	1057.2* (40.8)

EDL Po/body mass (milliNewtons/gram)	5.57 (0.19)	7.70* (0.35)	5.63 (0.13)	7.44* (0.14)

Costal diaphragm mass (milligrams)	252.2 (15.8)	290.5 (16.8)	242.9 (5.8)	260.9 (10.6)

Costal diaphragm mass/body mass (milligrams/grams)	1.70 (0.09)	1.80 (0.05)	1.64 (0.03)	1.84* (0.04)

All data are given as the mean with the standard error of the mean in parenthesis. * - statistically significant difference (p < 0.05) versus the appropriate vehicle control; # - statistically significant difference (p < 0.05) versus control animals (non-emphysemic).

#### Evaluation of muscle mass and Po following CRF2R agonist treatment

As can be seen in Table 2, absolute (+41%) and relative (+31%) EDL mass was significantly greater in Sham-PG873637 compared to Sham-vehicle animals while only relative EDL mass was significantly greater (+15%) in EMP-PG873637 compared to EMP-vehicle animals. Absolute (+48%) and relative (+38%) EDL Po was significantly greater in Sham-PG873637 compared Sham-vehicle animals. Absolute (+26%) and relative (+32%) EDL Po was significantly greater in EMP-PG873637 compared to EMP-vehicle animals. There was no significant difference in absolute and relative diaphragm mass in PG873637 treated compared to vehicle treated Sham animals. Relative (+12%) diaphragm mass was significantly greater in PG873637 treated compared to vehicle treated EMP animals.

### Rat CHF model

#### CHF induction

As can be seen in Table 3, all rats with a myocardial infarction demonstrate significant differences in fractional area contractions (66% decrease MI vehicle versus sham vehicle, 60% decrease MI PG873637 versus sham PG873637); ejection fraction (63% decrease MI vehicle versus sham vehicle, 58% decrease MI PG873637 versus sham PG873637); left ventricular systolic area (368% increase MI vehicle versus sham vehicle, 305% increase MI PG873637 versus sham PG873637); left ventricular systolic volume (465% increase MI vehicle versus sham vehicle, 447% increase MI PG873637 versus vehicle PG873637); left ventricular diastolic area (118% increase MI vehicle versus sham vehicle, 90% increase MI PG873637 versus sham PG873637); left ventricular diastolic volume (147% increase MI vehicle versus sham vehicle, 118% increase MI PG873637 versus sham PG873637); and lung weight (84% increase MI vehicle versus sham vehicle, 50% increase MI PG873637 versus sham PG873637) when compared to the appropriate sham operated rats. All differences were consistent with CHF including the greater lung weights. There were no major differences between rats treated with vehicle or the CRF2R agonist, PG873637, in any of the cardiac parameters.

**Table 3:** Effects of three months of treatment with either vehicle or PG873637 on sham operated or myocardial infracted rat body mass, fractional area contraction, ejection fraction, systolic area, systolic volume, diastolic area, diastolic volume, scar weight, lung weight, muscle mass and muscle absolute force (Po).

	Sham Vehicle 3 month	Sham PG873637 3 month	MI Vehicle 3 month	MI PG873637 3 month
Number of animals	7	7	10	9

Final body mass (grams)	482.8 (6.8)	476.8 (12.4)	480.5 (6.1)	487.4# (7.6)

Final Fractional Area Contractions (%)	63.4 (7.6)	65.2 (6.5)	21.7# (6.9)	26.0# (3.2)

Final Ejection Fraction (%)	68.2 (8.6)	72.6 (4.9)	25.3# (7.5)	30.5# (3.6)

Final LV Systolic Area (centimeters^2^)	18.5 (4.1)	17.7 (3.8)	86.7# (15.2)	71.8# (13.1)

Final LV Systolic Volume (milliliters)	0.2 (0.1)	0.2 (0.1)	1.3# (0.3)	1.0# (0.3)

Final LV Diastolic Area (millimeters^2^)	50.7 (5.0)	51.1 (5.2)	110.4# (14.7)	97.3# (18.5)

Final LV Diastolic Volume (milliliters)	0.7 (0.1)	0.7 (0.1)	1.7# (0.3)	1.5# (0.3)

Final Scar Weight (grams)	NA	NA	0.1 (0.0)	0.1 (0.0)

Final Lung Weight (grams)	1.5 (0.1)	1.5 (0.1)	2.8# (0.9)	2.2# (1.0)

EDL mass (milligrams)	189.9 (3.7)	228.6* (6.5)	191.0 (1.9)	230.0* (5.8)

EDL mass/body mass (milligram/gram)	0.41 (0.01)	0.47* (0.01)	0.40 (0.01)	0.47* (0.01)

EDL Po (milliNewtons)	3348.7 (78.9)	3909.9* (71.7)	3073.3# (96.3)	3927.7* (52.6)

EDL Po/body mass (milliNewton/gram)	7.16 (0.13)	8.09* (0.18)	6.41# (0.24)	8.12* (0.13)

Soleus mass (milligrams)	188.9 (3.2)	222.4* (7.9)	197.0 (2.6)	222.4* (7.9)

Soleus mass/body mass (milligram/gram)	0.40 (0.01)	0.46* (0.01)	0.41 (0.01)	0.45* (0.01)

Soleus Po (milliNewtons)	2033.6 (26.9)	2420.9* (88.1)	2177.5# (46.0)	2444.7* (68.8)

Soleus Po/body mass (milliNewton/gram)	4.38 (0.08)	4.99* (0.15)	4.53 (0.09)	5.06* (0.14)

All data given with standard error of the mean in paranthesis. * - statistically significant (p < 0.05) versus appropriate vehicle control: # - statistically significant (p < 0.05) versus appropriate sham control.

#### Evaluation of muscle mass and Po following CRF2R agonist treatment

Results of the analysis of EDL and soleus muscle mass and Po in sham and CHF rats treated with either vehicle or PG873637 are shown in Table 3. Analysis of the EDL muscle did not demonstrate a significant difference in either absolute or relative mass between the sham and MI vehicle treated rats; a statistically significant greater absolute and relative EDL mass was observed in both the sham (20% increase in absolute mass and 15% increase in relative mass) and MI (20% increase in absolute mass and 18% increase in relative mass) rats treated with PG873637 when compared to the appropriate vehicle control. Analysis of absolute and relative EDL muscle Po demonstrated a statistically significant lower Po in MI vehicle treated rats when compared to sham vehicle treated rats; absolute and relative EDL Po was significantly greater with PG873637 treatment in both sham (17% increase in absolute Po and 13% increase in relative Po) and MI (28% increase in absolute Po and 27% increase in relative Po) rats when compared to vehicle treatment. Analysis of absolute and relative soleus muscle mass demonstrated no difference between MI and sham vehicle treated animals; absolute and relative soleus mass was greater with PG873637 treatment in both sham (18% increase in absolute mass and 15% increase in relative mass) and MI (13% increase in absolute mass and 10% increase in relative mass) animals when compared to vehicle control. Analysis of absolute and relative soleus Po demonstrated a significantly greater absolute Po in MI vehicle treated rats compared to sham vehicle treated rats; treatment with PG873637 resulted in a significantly greater absolute and relative sham (19% increase in absolute Po and 14% increase in relative Po) and MI (12% increase in absolute Po and 12% increase in relative Po) soleus muscle Po compared to vehicle treated rats.

## Discussion

In this report we demonstrate for the first time that long term treatment with a CRF2R agonist maintains skeletal muscle mass and force production in animals with chronic disease (emphysematous hamsters and rats with CHF) and in aged rats.

### Aging Rat Model

Previously, we demonstrated that activation of the CRF2R can increase skeletal muscle mass and force production in young mice and rats [46-48]. In the present report we demonstrate that old rats, like young rats, respond to pharmacological activation of the CRF2R with greater absolute and/or relative skeletal muscle mass and force production when compared to age matched untreated rats. Thus, CRF2R functionality is maintained throughout the aging process. Interestingly, we observed that the aging-related loss of muscle mass and force was greater in the EDL muscle (predominantly Type II, fast twitch) than in the soleus muscle (predominantly Type I, slow twitch). This is similar to what has been observed by others [12,55,56]. The preservation of both Type I and Type II muscle CRF2R functionality in aged muscle indicates that in an aging associated physiological background, the CRF2R is functional and capable of maintaining muscle mass. Together, these data demonstrate that activation of the CRF2R can maintain muscle mass and force production and this is independent of aging associated physiological dysfunction such as nutritional imbalance, cumulative muscle damage, metabolic imbalance, alterations in hormones/cytokines and loss of regenerative potential [2-8],. A similar effect has been observed following treatment of aged F344 rats with the β2-adrenergic agonist, fenoterol [12]. Thus, treatment with a CRF2R agonist may be useful in combating the muscle weakness and frailty associated with aging.

### Hamster emphysema model

The purpose of this investigation was to determine whether the administration of a CRF2R selective agonist would maintain skeletal mass and force in animals with chronic EMP. The results of this investigation demonstrate that 5 months of treatment of EMP animals with a CRF2R selective agonist results in greater absolute and relative EDL force when compared to vehicle treated EMP animals. We did not see a difference in absolute EDL or diaphragm muscle mass in emphysemic animals treated with a CRF2R agonist when compared to vehicle treated EMP animals, although relative EDL and diaphragm mass were greater when compared to vehicle treated EMP animals.

#### Diaphragm atrophy

Similar to locomotory muscles the diaphragm is adaptable. It is well established that the diaphragm can adapt biochemically and structurally in response to alterations in metabolic load secondary to lung hyperinflation [24,28,33]. Specifically, EMP-induced increases in respiratory muscle metabolic demands [57] result in elevated oxidative enzyme capacity [24,28,33]. Moreover, EMP-induced diaphragm capillary proliferation is thought to be important for enhancing O_2 _diffusion and extraction [25]. In addition, the diaphragm muscle fibers shorten chronically which acts to re-establish a favorable position on the diaphragm length-tension curve [24,25]. In contrast, reports on EMP-induced diaphragm fiber cross-sectional changes are mixed. As discussed earlier, diaphragm fiber hypertrophy, atrophy, or no change has been reported in animal models of EMP. Respiratory muscle weakness is well documented in COPD patients and can lead to hypercapnia [23,58-61]. Thus, if respiratory muscle weakness contributes to chronic hypercapnia and ultimately increased mortality in COPD patients, it is of the utmost importance to find therapeutic interventions aimed at preventing this negative outcome. In this investigation, we did not observe significant changes in diaphragm muscle mass in EMP animals although significant changes in lung and right ventricles did occur. Nor did we observe significant changes in EDL muscle mass or force production in EMP animals compared to control animals. Importantly, treatment with a CRF2R agonist resulted in a directional increase in diaphragm muscle mass in both normal and EMP animals and a significant increase in EDL force production in normal and EMP animals. Interestingly, EDL muscle mass increased with CRF2R agonist treatment in normal animals but not in EMP animals. At present we do not understand the significance of the disconnect between EDL mass and force in EMP animals but future studies will focus on understanding this observation.

#### Mechanical ventilation

Acute respiratory failure (ARF) in COPD patients is a common cause of hospital admissions [62]. Mechanical ventilation (MV) is often administered if conservative treatment fails [63,64]. However, mortality rates rise from 10% for conservative treatment up to 50% if MV is required [65]. Moreover, weaning from MV is clinically challenging, with up to 67% failure rate in COPD patients with respiratory muscle weakness and/or dysfunction [66-69]. Those patients that do survive spend a longer time weaning in comparison to most other pathological conditions [70]. Extubation failure from MV occurs in other conditions, with up to 20% of all patients on prolonged MV experience weaning difficulties [71]. Although the mechanisms for these difficulties are not fully understood, respiratory muscle dysfunction contributes to this problem [72,73]. Indeed, diaphragm fatigue has been demonstrated as a major factor affecting weaning failure [74,75]. For example, Le Bourdelles et al. demonstrated that mechanically-ventilated animals have reductions in diaphragm isometric force and mass [76]. Furthermore, Powers and coworkers have extended this work by demonstrating prolonged MV promotes increased diaphragm protein degradation, reduced protein synthesis, increased oxidative stress, and impairment of antioxidant defenses [72,77,78]. Therefore, conservation of respiratory muscle mass may reduce MV treatment length and weaning difficulties. The results of this investigation provide suggestive evidence that CRF2R agonist may increase diaphragm muscle mass. This together with the observation that treatment with a CRF2R agonist is effective in preventing loss of non-actively contracting skeletal muscle [46,48] suggest that treatment with a CRF2R agonist may be an important therapeutic intervention for ameliorating the deleterious effects of MV on diaphragm mass and function. More work will be needed before we fully understand the potential of CRF2R agonist in treating MV weaning failure.

### Rat CHF model

In this report we have evaluated the effect of CRF2R agonist treatment on skeletal muscle mass and function in rats with ongoing CHF. Treatment with a CRF2R agonist resulted in greater EDL (fast twitch) and soleus (slow twitch) absolute and relative skeletal muscle mass and force production in rats with and without chronic CHF when compared to the appropriate vehicle treated animals. Interestingly, the EDL muscle demonstrated lower absolute force production in CHF rats when compared to sham rats without a significant difference in mass; no difference in absolute force production or mass was observed in the soleus muscle from CHF rats. Even with this deficit in EDL function, the CRF2R mediated difference in mass and force production was similar in both normal and CHF rats when compared to vehicle treated normal and CHF rats, indicating that CRF2R functionality was not compromised by the changes that cause a loss in force. The observation that oxidative muscles are more resistant than glycolytic muscles to loss of function in CHF has been observed previously in mice [79,80]. In addition, the loss of skeletal muscle force but not mass has been described previously both in animals and humans with CHF, although there is model and species specificity in the type of muscle fiber that is affected [44].

CHF results in major physiological dysfunction at both the organismal and tissue levels including cardiac cachexia. These changes are believed to result from decreased tissue oxygenation and increased levels of stress hormones and cytokines including TNF-α, IL-6 and cortisol [34-36]. Even though these catabolic cytokines/hormones are present, activation of the CRF2R in CHF rats resulted in increased muscle mass and force production, indicating that the CRF2R pathway is able to override these catabolic signals. Thus, treating CHF patients with a CRF2R agonist may be a useful therapeutic option to improve strength and muscle function in patients suffering from cardiac cachexia.

### Overall Significance

Together the findings described in this report provide support for the concept that pharmacological activation of the CRF2R may prove useful in improving muscle function in individuals experiencing weakness associated with chronic disorders and aging. The most consistent finding amongst all three models is the observation that CRF2R agonist treatment results in greater muscle force production compared to vehicle treatment. The change in force production observed with CRF2R agonist treatment was often, but not always, associated with a change in muscle mass - the exception being emphysemic hamsters (although when the change in muscle mass is normalized to body mass a significant change is observed). The reason for this discontinuity is at present unclear since in our previous investigations of acute and chronic atrophy (cancer cachexia and disuse atrophy), we consistently observed parallel increases in absolute muscle mass and force production following CRF2R activation [46-51]. Additional work is necessary to determine if this exception is significant or an experimental artifact.

An interesting observation from this work is that even though skeletal muscle weights were increased following CRF2R activation, increases in absolute body mass were not consistently observed. This is puzzling since skeletal muscle accounts for a significant portion of absolute body mass. This inconsistency can be explained by the known effects of CRF2R activation on another major component of absolute body mass, adipose tissue. Recent reports have shown that the CRF2R has a major role, via both central and peripheral action, in modulating body mass [81-84]. Importantly for this report, it has been observed that peripherally administered CRF2R agonists both increases skeletal muscle mass as well as decreases adipose tissue mass in normal rats [81], most likely by direct activation of CRF2Rs in skeletal muscle and adipose tissue. Thus, the net effect of CRF2R activation on absolute body mass depends on the relative amounts of skeletal muscle and adipose tissue in an animal. We have observed that when skeletal muscle makes a greater contribution to absolute body mass than adipose tissue (such as in lean young animals), CRF2R activation results in an increase in body mass; we have also observed a decrease in body mass following CRF2R activation when the adipose tissue content of an animal is high (obese animals) and the resulting loss of adipose tissue mass more than offsets the gain in skeletal muscle mass (RJI, unpublished observations).

Finally, we believe the most significant aspect of this work is the observed robustness of the effect of CRF2R activation on skeletal muscle mass and force production. The findings in this study, when combined with our previous findings, demonstrate that activation of the CRF2R results in greater skeletal muscle mass and force production compared to vehicle treatment and that the CRF2R mediated changes are independent of specie, physiological condition, treatment length and dosing paradigm [46-51]. In addition, the observation that chronic administration of a CRF2R agonist maintains muscle mass and force production during the entire treatment period demonstrates that down regulation of the CRF2R does not occur with continuous stimulation. This provides evidence that chronic administration of a CRF2R agonist will result in long term maintenance of muscle function in patients suffering from chronic disease. Collectively, these studies provide the rationale for considering CRF2R agonists as potential therapeutics for the treatment of both acute and chronic muscle wasting disorders in humans.

## Conclusion

We found that treatment of aged animals and animals with a chronic disease with a CRF2R agonist resulted in maintenance of skeletal muscle mass and force production when compared to vehicle treatment.

## Competing interests

This work was supported by the Procter & Gamble Company. RTH, FRL, ETD, DLR, JMZ, TPO, KGM and RJI are or were at the time these studies were performed employees of the Procter & Gamble Company.

## Authors' contributions

All authors made substantial intellectual, design and/or executional contributions to this study as follows: RTH provided intellectual, design and executional contributions; FRL provided executional contributions; ETD provided executional contributions; DLR provided executional contributions; JMZ provided design and executional contributions; TPO provided intellectual and design contributions; KGM provided executional contributions; JPM provided intellectual, design and executional contributions; LFF provided executional contributions; TIM provided intellectual and design contributions; DCP provided intellectual and design contributions; and RJI provided intellectual, design and executional contributions. All authors gave final approval of the manuscript.

## Pre-publication history

The pre-publication history for this paper can be accessed here:

http://www.biomedcentral.com/1471-2474/12/15/prepub
